# Advancements in High-Resolution Computed Tomography: Revolutionising Bone Health Micro-Research

**DOI:** 10.3390/bioengineering12111189

**Published:** 2025-10-31

**Authors:** Richard Lindtner, Lukas Kampik, David Putzer, Miranda Klosterhuber, Anton Kasper Pallua, Werner Streif, Michael Schirmer, Gerald Degenhart, Rohit Arora, Johannes Dominikus Pallua

**Affiliations:** 1 Department of Orthopaedics and Traumatology, Medical University of Innsbruck, Anichstraße 35, 6020 Innsbruck, Austria; richard.lindtner@i-med.ac.at (R.L.); lukas.kampik@i-med.ac.at (L.K.); david.putzer@i-med.ac.at (D.P.); miranda.klosterhuber@i-med.ac.at (M.K.); rohit.arora@i-med.ac.at (R.A.); 2 Former Institute for Computed Tomography-Neuro CT, Medical University of Innsbruck, Anichstraße 35, 6020 Innsbruck, Austria; anton.k.pallua@cnh.at; 3 Department of Pediatrics I, Medical University of Innsbruck, Anichstraße 35, 6020 Innsbruck, Austria; werner.streif@i-med.ac.at; 4 Office Dr. Schirmer, 6060 Hall, Austria; schirmer.michael@icloud.com; 5 Core Facility MicroCT, University Clinic for Radiology, Medical University of Innsbruck, Anichstraße 35, 6020 Innsbruck, Austria; gerald.degenhart@i-med.ac.at

**Keywords:** bone health, micro-computed tomography, high-resolution peripheral quantitative computed tomography, finite element analysis, machine learning

## Abstract

Bone disorders such as osteoporosis, osteopenia, and osteoarthritis affect millions worldwide, creating an urgent need for earlier and more accurate assessment of bone health. High-resolution imaging has transformed this field: micro-computed tomography remains the research gold standard for ex vivo and preclinical studies, while high-resolution peripheral quantitative computed tomography enables in vivo clinical assessment of trabecular and cortical bone microarchitecture. Combined with finite element analysis and machine learning, these modalities enable biomechanical modelling, predictive risk stratification, and progress toward personalised treatment. Remaining challenges include cost, limited availability, motion artefacts, and lack of standardisation. This review summarises current advances in imaging and computational methods, highlights their respective strengths and limitations, and outlines future directions required to translate these technologies into routine clinical practice.

## 1. Introduction

Bone health is central to mobility, organ protection, and mineral storage. Disorders such as osteoporosis, osteoarthritis, and Paget’s disease of bone impair skeletal integrity through distinct mechanisms: reduced bone mass, joint degeneration, and disorganised remodelling. All of these conditions increase fracture risk and impose significant personal and socioeconomic burdens [1,2,3,4,5]. Accurate assessment of bone quality, including density, microarchitecture, and mechanical competence, is therefore essential for early diagnosis, risk prediction, treatment evaluation, and implant fixation. Recent advances in imaging have transformed bone research. Micro-computed tomography (micro-CT) provides ex vivo high-resolution morphometry of bone specimens, offering micrometre-scale detail that cannot be achieved in vivo due to radiation dose and scanning time constraints [6]. In contrast, high-resolution peripheral quantitative computed tomography (HR-pQCT) enables in vivo assessment of trabecular and cortical bone microstructure at peripheral sites with clinically acceptable resolution and radiation exposure [7]. Both are now central to understanding bone structure, pathology, and treatment effects. CT technology has evolved from millimetre-scale medical imaging to micrometre-scale micro-CT and synchrotron-based nano-CT, enabling detailed three-dimensional (3D) morphometry and even time-resolved studies [8,9,10,11,12,13,14,15,16,17]. These advances support both basic research and translational applications in bone biology. Parallel to imaging advances, machine learning (ML) has evolved rapidly. Convolutional neural networks (CNNs) and deep learning architectures such as U-Net now enable automated segmentation, artefact correction, and predictive modelling in CT datasets [18,19,20,21,22,23]. This review summarises advances in high-resolution imaging (micro-CT, HR-pQCT) and their integration with finite element analysis (FEA) and ML. We highlight applications in bone health, discuss strengths and limitations, and outline future research needs and clinical translation.

## 2. X-Ray-Based High-Resolution CT Modalities

To avoid conflating preclinical and clinical systems, this section is organised from ex vivo to in vivo: laboratory micro-CT, synchrotron radiation (SR) micro-CT, and clinical derivatives (pQCT/HR-pQCT). For each modality, (i) principles and system types, (ii) typical applications, and (iii) strengths and limitations are outlined, and indications of how image volumes feed into FEA and machine-learning workflows are provided.

### 2.1. Laboratory Micro-CT

#### 2.1.1. Principles and System Types

**Figure 1:**
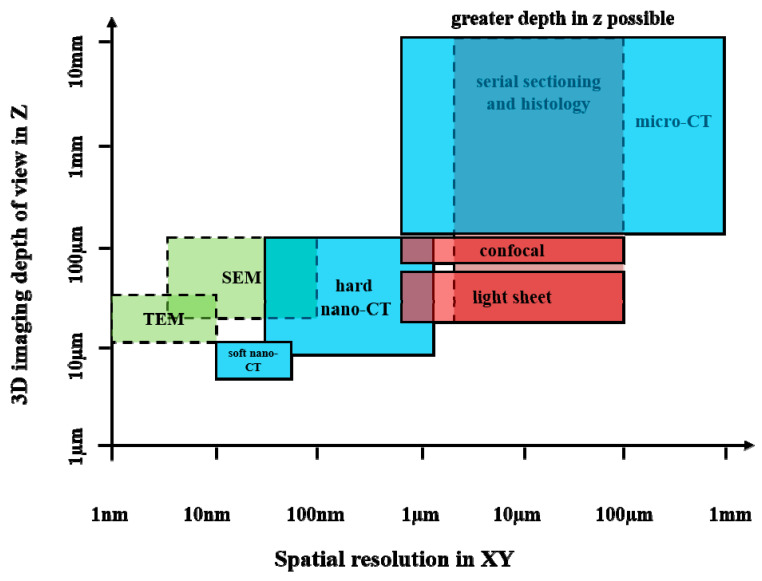
An overview of the spatial resolution (in XY) and full depth (in Z) of various 3D imaging techniques for life sciences applications is depicted. The methods are categorised according to colour: blue for CT, green for EM, and red for LM techniques. The solid line indicates non-invasive methods, while the dashed line indicates destructive ways. The techniques include TEM, scanning electron microscopy (SEM), soft nano-CT, hard nano-CT, and micro-CT—data adapted from [10,33,34,35,36,37].

Laboratory micro-CT utilising X-rays’ penetrating power enables non-invasive imaging of a large field of view (FoV)—even for optically opaque materials—across a range of resolutions (as shown in Figure 1), with comparatively straightforward sample preparation [10]. In contrast, much of our understanding of the relationship between structure and functionality in biology is derived from various 2D imaging tools, including optical and transmission electron microscopy (TEM) [24]. These tools have benefited from the significant increase in available labels and markers that enable the identification of specific features [25]. While light microscopy (LM) and electron microscopy (EM) workflows can achieve partial 3D (e.g., confocal, light-sheet, serial sectioning) [26], they are limited by optical transparency requirements [27], demanding sample preparation (e.g., freezing, fixing, embedding in resin, and serial sectioning) [28], and a limited FoV (as in TEM) [29].

Compared to medical CT, micro-CT offers voxel sizes up to orders of magnitude smaller and resolutions down to ~1 μm. It typically employs cone-beam geometry with a rotating sample between a fixed source and detector, enabling superior resolution at the cost of higher radiation doses [30,31,32].

**Figure 2:**
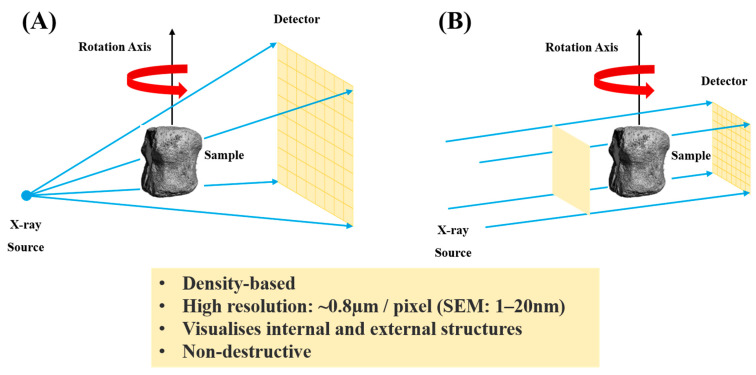
Schematic representation of configurations for micro-CT: (**A**) A cone beam system is often used in laboratory settings and typically employs a single X-ray source and detector. (**B**) SR X-ray systems typically use parallel-beam geometry—data adapted from [12].

Measurement geometry contrasts: In medical CT, the detector and source rotate around a fixed patient table, often using fan-beam geometry. In contrast, lab micro-CT typically employs a rotating sample holder with a fixed detector and source (cone-beam geometry) [38]. Using high-energy X-rays and extended exposure durations in micro-CT allows for superior spatial resolution compared to standard CT imaging of patients [39]. However, some small-animal micro-CT systems use fixed sample holders [40]. Figure 2 shows configurations for micro-CTs.

#### 2.1.2. Applications in Life Sciences

Scope and positioning: Biomedical imaging involves examining biological samples or entire animals in vivo, ex vivo, or in situ rather than diagnosing patients in a hospital setting. While optical microscopy is commonly used for this purpose, micro-CT has become a powerful and versatile tool that can either supplement or replace other imaging modalities due to the ability of X-rays to penetrate deeply into matter, allowing for rapid visualisation of 3D structures. Micro-CT can perform “digital histology” by generating large virtual sections simultaneously, facilitating more efficient analysis than conventional histopathological slicing [10,12,13].

**Figure 3:**
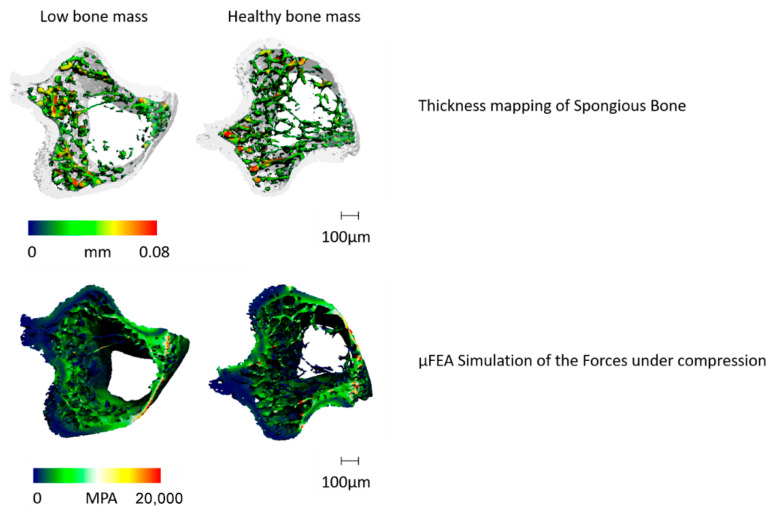
Micro-CT analysis of a mouse tibia specimen. Top: Local thickness distribution maps illustrating the microstructural organisation of trabecular bone. Bottom: Finite element-based stress analysis showing regional variations in mechanical loading capacity. Scale bar = 100 µm.

Calcified tissues (bone, teeth): When imaging calcified biological tissue such as bone, attenuation contrast CT is typically used because the high X-ray attenuation of calcium provides high contrast. This technique enables the quantitative imaging of bone structure, porosity, and mineral density, which can be used to study relationships between bone morphology [41] and biological factors, such as ageing [40] and drug use [41], or to determine the post-mortem interval [42,43]. An example is shown in Figure 3, where a micro-CT reconstruction of a mouse tibia specimen illustrates structural and mechanical characterisation at high resolution. The upper panel shows local thickness maps, while the lower panel displays finite element-based stress distribution, highlighting the ability of micro-CT not only to visualise trabecular architecture but also to provide biomechanical insights.

Additional applications and exemplars (hard tissues): Micro-CT has been widely used to map porous structures, lesions, fractures, and regeneration in hard tissues. For example, tomograms of corals reveal channels crucial to understanding directional growth [44]. Micro-CT of primate molar teeth can determine their displacement under load, providing insight into the relationship between the morphology of the periodontal ligament space and tooth movement [45,46]. Micro-CT has also been used to demonstrate the effects of bone ageing on the enlargement of Haversian canals in human cortical bone, contributing to a better understanding of the causes of bone thinning [47,48,49]. Additionally, micro-CT has been used to study bone regeneration in the rat tibia [50], trabecular bone mass degeneration in mice [46], the metabolic control of collagen synthesis in bone junctions [51,52], and the complex lacuna-canalicular network (LCN) structure within bone [53].

Non-calcified tissues (contrast-enhanced): The micro-CT technique proves highly useful for non-calcified biomedical samples, which generally exhibit weak intrinsic X-ray attenuation. With contrast agents (heavy-element liquids, gases, nanoparticles), even these biosamples benefit from micro-CT through increased attenuation and enhanced tissue contrast [54,55,56,57,58,59,60,61,62]. Micro-CT has been used to analyse numerous soft tissues, generating 3D anatomical data for mutant and experimental phenotypes [63]. For example, tomograms of stained hearts and lungs enable observation of fibrous muscle orientation and blood vessels [64]. This imaging method has also been utilised to map the neuronal architecture of animal brains, providing insight into the spatial organisation and connectivity [65,66]. In food science, the characterisation of meat as soft tissue has been used to study the distribution and amount of fat in products such as salami, beef, and lamb, which impact taste and texture [67].

Disciplinary breadth: Micro-CT has broad applications across numerous medical and scientific disciplines [38]. In addition to its medical applications, micro-CT is employed in industrial imaging, biology, and geology. It enables the analysis of material structures, including fractures and manufacturing defects, and facilitates the study of geological formations and fossils [38]. Despite the continuous improvement in image resolution and potential therapeutic implications, awareness of micro-CT’s capabilities among clinicians remains limited [68]. As a versatile preclinical analogue of clinical CT, micro-CT offers higher spatial resolution for imaging small animal models and various specimens. As technology advances, the applications and impact of micro-CT are expected to expand across multiple medical fields and beyond, fostering scientific and technological innovation [12,13,68,69,70,71,72,73].

#### 2.1.3. Strengths and Limitations of Micro-CT

Strengths: Laboratory micro-CT provides micrometre-scale, isotropic 3D resolution with high signal-to-noise, enabling quantitative morphometrics (e.g., BV/TV, Tb.N, Tb.Th, Tb.Sp), porosity analysis, and mineral-density estimation on the same volume [6]. Reconstructions are naturally suited for downstream computational pipelines such as FEA and machine-learning-based segmentation or phenotype discovery [74]. Sample preparation is comparatively straightforward for calcified tissues, and the technique supports a wide range of specimen sizes and materials [51]. With appropriate staining or contrast agents, soft-tissue microarchitecture can also be visualised (“digital histology”), allowing multi-scale correlation with optical or electron microscopy [54].

Limitations: FoV and gantry/sample-stage geometry constrain the maximum specimen size and shape, and there is an inherent resolution–specimen-size trade-off [52]. Image quality may be affected by beam hardening, ring and motion artefacts, partial-volume effects at thin structures, and threshold-selection bias during segmentation [75]. Scan time and radiation dose—particularly for in vivo small-animal protocols—require careful optimisation and standardised quality control for longitudinal studies [76]. For soft-tissue applications, contrast enhancement can introduce preparation-related changes (e.g., shrinkage or uneven staining) and requires protocol harmonisation [54]. Metal or highly attenuating inclusions can cause streak artefacts that complicate quantitative analysis [77]. Cross-scanner comparability depends on calibration and reporting standards (e.g., voxel size, energy, filtration, reconstruction, and segmentation parameters), which should be documented to ensure reproducibility and enable meta-analyses [6].

#### 2.1.4. Comparison with Other Imaging Modalities

To contextualise micro-CT/HR-pQCT within preclinical and clinical imaging, we added a cross-modality comparison (Table 1). MRI provides excellent soft-tissue contrast and marrow characterisation; ultra-short echo-time (UTE/ZTE) sequences allow limited cortical signal but not trabecular-scale morphometry at clinical field strengths [78]. PET-CT offers molecular sensitivity (e.g., bone turnover, metabolism, infection) but lower spatial resolution; the CT component supplies anatomy and attenuation correction [79]. Ultrasound resolves superficial soft tissues and cortex interfaces in real time without ionising radiation, but acoustic impedance mismatch prevents imaging through cortical bone, limiting intraosseous assessment [80]. In contrast, micro-CT (ex vivo) and HR-pQCT (in vivo) uniquely quantify 3D bone microarchitecture and support FE analysis for mechanical competence [6].

#### 2.1.5. Special Topic: Micro-CT in Paleoradiology and Evolutionary Biology

**Figure 4:**
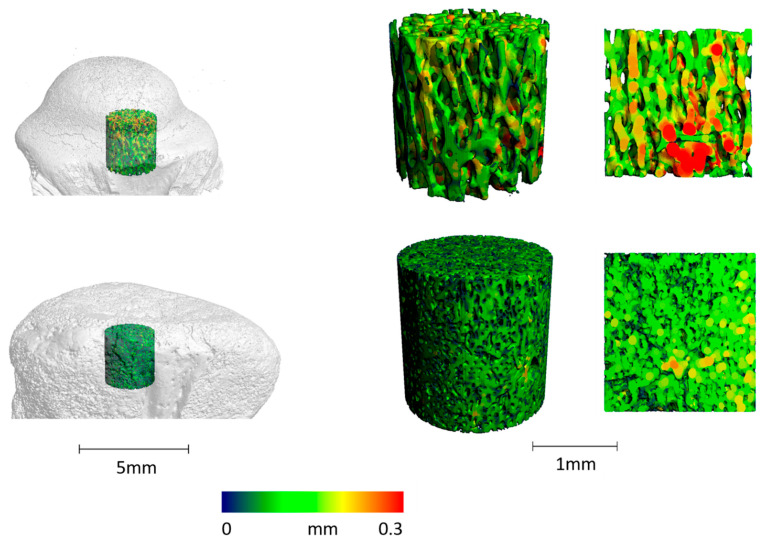
Three-dimensional micro-CT reconstructions of trabecular bone microstructure from a fossilised vertebra of *Champsosaurus* sp. (70–73 million years old; top row) compared to a modern Crocodylidae vertebra (bottom row). (**Left**) cylindrical region of interest (ROI) extracted from the vertebral centrum (scale bar = 5 mm). (**Middle**) 3D rendering of trabecular architecture within the ROI, colour-coded by local thickness (scale bar = 1 mm). (**Right**) magnified view of trabecular details illustrating heterogeneity in bone thickness, with the colour map ranging from thin (blue/green) to thick (yellow/red) trabeculae. Data adapted from [41].

Paleoradiological studies have demonstrated that micro-CT imaging can provide highly detailed insights into the microstructure of fossilised bone specimens, enabling comparisons across vast evolutionary timescales. For example, three-dimensional analyses with local thickness and separation measurements [84] reveal the microstructural characteristics of a fossilised vertebra from *Champsosaurus* sp., estimated to be 70–73 million years old. These findings can be directly compared with those from modern Crocodylidae vertebrae, as illustrated in Figure 4.

#### 2.1.6. Recent Desktop-Scanner Advances (Ex Vivo)

Ex vivo imaging has made considerable progress in achieving sub-micron resolution for larger specimens while reducing scan time. This has enabled bone scientists to conduct sophisticated analyses using desktop scanners, which were previously only available to SR micro-CT users. Advances in clinical and research imaging are expected to enhance understanding of osteoporosis-related skeletal issues and support targeted treatments [71].

### 2.2. SR Micro-CT

#### 2.2.1. Principles

SR micro-CT exploits the high brilliance, partial coherence, tunability, and low divergence of synchrotron X-ray beams to achieve sub-micrometre, isotropic 3D resolution with excellent contrast and quantitative fidelity. In contrast to laboratory cone-beam systems, SR micro-CT typically uses a monochromatic, quasi-parallel beam selected by a double-crystal monochromator, which mitigates beam-hardening artefacts and stabilises linear attenuation measurements for small specimens [85]. In a standard SR micro-CT setup, a parallel beam traverses the specimen and is recorded by a scintillator–objective–camera detector chain, where optical magnification sets the effective pixel size. High photon flux enables short exposures and high signal-to-noise ratio, while flat-fielding and ring-artefact suppression are applied prior to parallel-beam reconstruction [86]. Because synchrotron beams are partially coherent, SR micro-CT readily supports phase-contrast modes—especially propagation-based (inline) phase contrast—which enhance edges and weakly absorbing structures. Single-distance phase retrieval and modern multi-material/regularised schemes convert phase contrast to quantitative maps and improve spatial resolution in the tomographic volume [87]. Energy tunability further enables spectral methods such as K-edge subtraction (KES), allowing material-specific contrast and reducing ambiguity between similarly absorbing species; recent work demonstrates high-resolution KES for tracking cells/scaffolds in vivo and vascular applications [88]. These physical advantages explain SR micro-CT’s widespread use in musculoskeletal research—resolving lacuno-canalicular networks, micro-damage, and mineral heterogeneity that are below the voxel sizes of clinical imaging—while providing data suited to quantitative analyses and advanced modelling [89].

#### 2.2.2. Bone Applications

In bone and biomaterials research, SR micro-CT has been applied to trabecular architecture, micro-damage mapping, scaffold morphologies, mineralisation patterns, adaptation to disuse, the bone–implant interface, a variety of bone/biomaterial systems, and osteocyte lacunar properties, including site-specific mineral distributions [90]. At this resolution, datasets support qualitative and quantitative analyses of vasculature and lacunar voids, providing detailed microstructural insight [91]. These investigations are conducted ex vivo on small-sized bone specimens [89]. SR micro-CT is highly suitable for 3D non-destructive evaluation of small-sized bone specimens ex vivo [71]. The technology has been used to investigate various bone characteristics, such as trabecular architecture [92], micro-damage [93,94,95,96,97,98], bone-tissue scaffold morphologies [99,100], mineralization [96,101,102,103], bone adaptation to disuse [104], bone–implant interface [105,106,107], bone and biomaterials [108,109], and osteocyte lacunar properties and site-specific mineral distributions [101,110,111,112,113,114,115,116,117]. The high resolution of SR provides both qualitative and quantitative data on bone vasculature and lacunar voids [71], opening opportunities to investigate new research areas non-invasively in bone samples of reasonable size.

#### 2.2.3. Strengths and Limitations of SR Micro-CT

Strengths: SR micro-CT delivers sub-micrometre, isotropic 3D resolution with high partial coherence and monochromatic, quasi-parallel illumination, which reduces beam-hardening artefacts and stabilises quantitative attenuation, yielding high SNR datasets suited to advanced morphometrics and modelling [118]. The beam’s tunability enables phase-contrast modes (propagation-based) for edge enhancement of weakly absorbing features and phase retrieval to convert phase to quantitative maps; K-edge subtraction further allows material-specific contrast [119]. High flux and optics (scintillator–objective–camera) support optical magnification down to ~0.3–1.0 µm voxel sizes for micro-CT (and below for nano-CT), enabling quantitative analysis of trabeculae, microdamage, and the LCN [120]. SR micro-CT is also compatible with in situ mechanical testing protocols, linking evolving microstructure with deformation and crack propagation for mechanistic insight [121].

Limitations: For bone, SR micro-CT is predominantly ex vivo because in vivo doses remain prohibitive at the resolutions of interest [89]. FoV and scanable volume are limited by beamline geometry and optics, constraining sample size and the ability to image whole human bones at high resolution [43]. Access is restricted, and datasets are computationally heavy, requiring specialised analysis workflows [122]. Radiation exposure can alter tissue, necessitating careful dose management, especially during repeated scans [123]. Phase-contrast imaging requires phase retrieval, which introduces method-dependent trade-offs and can bias fine-scale quantitation if misapplied [119]. Imaging is still susceptible to ring artefacts and related detector issues, requiring dedicated correction to preserve quantitative fidelity [124].

### 2.3. High-Resolution Peripheral Quantitative Computed Tomography (HR-pQCT)

#### 2.3.1. Principles and System Evolution

The advent of peripheral quantitative computed tomography (pQCT) and its high-resolution counterpart, HR-pQCT, has marked a significant milestone in bone health research. These technologies have provided unprecedented insights into the microarchitecture of bones, enabling more accurate assessments of bone quality and strength.

pQCT developed as a promising tool for identifying disease risk factors, such as osteoporosis, concurrently with the laboratory development of micro-CT [125]. DXA, one of the most widely used clinical methods, cannot accurately identify patients at risk of fracture; however, pQCT offers an improved method for assessing bone clinically [126,127]. While having a lower resolution than micro-CT, pQCT enabled non-invasive, time-lapse imaging of patients. The resolution of pQCT made it impossible to accurately examine bone microarchitecture in individuals with degenerative bone diseases (typical trabecular thickness ~200 µm), hindering direct comparability with micro-CT [128,129]. HR-pQCT devices were introduced as enhanced replacements for pQCT to address this limitation. Direct comparison to micro-CT became feasible with isotropic voxel sizes of 82 and 61 µm (XtremeCT I/II, Scanco Medical) [72]. HR-pQCT, introduced in 2005, achieves voxel sizes of 82–61 µm while maintaining low radiation exposure (~5 µSv) [130,131,132]. Unlike DXA, it provides 3D insight into trabecular and cortical bone, enhancing fracture risk assessment. Integration with FEA allows biomechanical evaluation of bone strength [133,134].

#### 2.3.2. Reporting and Metrics (Harmonised with Micro-CT)

Micro-CT scanners, first developed and introduced by Feldkamp in the 1980s, have become standard tools in bone research [71]. They are widely used to evaluate the 3D morphologies of bone structures at image resolutions suitable for bone samples ranging from small animals to human bone biopsy specimens. These scanners correlate well with 2D histological data for trabecular thickness, number, separation, and the bone volume-to-total volume ratio (BV/TV) [135,136,137]. While histomorphometric measurements enable the study of both static and dynamic cellular properties, micro-CT can rapidly and reliably quantify 3D bone structural morphology data in a non-destructive manner. Studies have included quantifying subchondral changes in guinea pigs [138], investigating bone morphology for growth [139], analysing skeletal characteristics in various mouse strains [136,140,141,142,143,144,145] and genetically altered mouse models [141,146,147], examining animal models of postmenopausal osteoporosis [137,145,148,149], assessing skeletal effects from mechanical loading [150] or unloading/disuse [140,151,152], studying microdamage [153] and fracture healing [154,155,156], observing longitudinal changes in the trabecular structure of biopsies from pre- and postmenopausal women [135], investigating pharmacological treatments [148,157,158], and examining bone implants [159] and engineered tissue in scaffolds [160,161].

Standards: To ensure comparability across pQCT/HR-pQCT (and micro-CT where applicable), recommended variables include trabecular BV/TV, Tb.N, Tb.Th, Tb.Sp; cortical Tt.Ar, Ct.Ar, Ct.Ar/Tt.Ar, Ct.Th, Ct.Po [111,162]. In addition, SMI and degree of anisotropy (DA) are sometimes reported, but SMI is sensitive to resolution and segmentation and should be interpreted cautiously in in vivo HR-pQCT. SMI reflects plate- vs. rod-like trabeculae; DA quantifies preferential orientation. Both are relevant for FEA, influencing directional mechanical competence, fracture-risk prediction, and implant anchorage [163,164]. Table 2 (below) summarises core metrics. Additionally, appropriate image resolutions and segmentation thresholds are crucial for reliable structural and density estimates (apparent and tissue), often calibrated with hydroxyapatite standards [164,165].

Beyond classical morphometrics: Recent studies emphasise graph- and topology-based descriptors that capture higher-order connectivity and organisational “blueprints” of trabecular networks. Alterations in such topological patterns have been associated with skeletal pathology and may augment strength and risk modelling when combined with FEA and density mapping. Incorporating these descriptors alongside conventional metrics could improve sensitivity to disease-related microarchitectural changes [166].

Note on FE pipelines: Standard micro-CT systems have also facilitated the development of mathematical models and FE analysis of bone tissue architecture and density, which can predict the stress and strain that may cause microdamage under specific mechanical loading conditions [167,168]. By analogy, HR-pQCT volumes, despite coarser resolution, are widely used for subject-specific FEA to estimate stiffness/strength in vivo.

#### 2.3.3. Clinical Applications and Integration with FE/ML

HR-pQCT applications include osteoporosis research, fracture healing [169], implant assessment [170], and evaluation of systemic diseases [171,172]. When coupled to FEA and machine-learning models that incorporate clinical covariates and robust QC, HR-pQCT supports predictive risk stratification and individualised treatment planning.

#### 2.3.4. Surgical Planning, Density Mapping, and Robotics

In surgical applications, pQCT and HR-pQCT provide valuable information for preoperative planning by mapping local variations in cortical and trabecular bone density. This enables optimisation of implant placement and screw positioning in areas with higher bone quality, particularly important in osteoporotic patients. Such targeted anchorage improves primary stability, reduces the risk of implant loosening, and contributes to better outcomes. By integrating pQCT-derived density profiles into surgical workflows, procedures such as joint replacement or robotic-assisted interventions (e.g., MAKO systems) can be tailored to the patient’s individual bone characteristics [7,169,170]. The primary structural formations in animals are composed of both soft (non-mineralised) and hard (mineralised) tissues. Hard tissues, such as bones and teeth, are stiff due to tissue mineralisation, while soft tissues, including muscles, ligaments, and tendons, exhibit elasticity and may be filled with aqueous solutions, such as those found in internal organs [173]. Micro-CT enables structural assessment across this continuum—from functionality and development to lesions, disease progression, fractures, and regeneration [13].

## 3. Computational Methods: Finite Element Analysis and Artificial Intelligence

Additionally, HR-pQCT data can be utilised for finite element analysis (FEA) to simulate mechanical properties and predict fracture risk, thereby providing a biomechanical perspective on bone health assessments. HR-pQCT data can be integrated into predictive models for bone fracture risk by using its detailed microarchitectural measurements as inputs to computational models that simulate bone strength and predict fracture susceptibility. FEA is one approach that uses HR-pQCT data to model mechanical properties and assess the impact of microstructural changes on bone integrity [174]. Additionally, integrating HR-pQCT measurements with clinical data, such as patient demographics and medical history, allows for the development of comprehensive models that account for various risk factors. These models can be enhanced using machine learning algorithms to identify patterns and correlations that may not be evident through traditional statistical methods, ultimately leading to more accurate and individualised fracture risk assessments [175]. Despite its advantages, HR-pQCT faces several challenges, including its high cost, limited availability in clinical settings, and the need for specialised training for image interpretation.

Additionally, motion artefacts can degrade image quality, affecting measurement precision. Machine learning algorithms, notably convolutional neural networks, have recently advanced, showing promise in reducing motion artefacts and increasing the accuracy of HR-pQCT measurements [176]. There is also a need for standardised protocols to ensure consistency and comparability across studies. To contextualise these advances, the following subsections outline a standardised FEA workflow and provide specific AI application examples that are already used or directly translatable to HR-pQCT and micro-CT pipelines [174,175,176].

### 3.1. Finite Element Analysis

Finite element analysis (FEA) is a computational technique for simulating bone behaviour under loading. Using high-resolution data (micro-CT, HR-pQCT), it predicts stress distribution, fracture risk, and implant performance. FEA is a computational technique used to simulate and predict the mechanical behaviour of structures under various loading conditions [177]. When applied to bone micro-CT data, FEA provides a powerful means of assessing bone strength and fracture risk by modelling the biomechanical properties of bone tissue [178,179]. Micro-CT offers detailed images of bone, serving as the basis for creating finite element models. It predicts stress distribution and identifies weak points under load [180]. This analysis offers insights into the mechanical competence of bone, enabling the assessment of its structural integrity [181]. FEA supports personalised treatment planning and implant design [182,183,184,185]. FEA plays a crucial role in studying bone diseases, such as osteoporosis, where alterations in bone microarchitecture have a significant impact on mechanical properties. It provides a detailed understanding of how these changes affect bone strength and contribute to an increased risk of fractures, aiding in the development of targeted therapeutic strategies [186]. In orthopaedic research, FEA designs and tests implants by analysing their interaction with bone. It helps optimise implant shapes and materials to ensure proper load transfer and minimise stress shielding, enhancing implant longevity and performance [187,188,189]. Creating accurate finite element models requires high-quality micro-CT data and sophisticated computational resources. The complexity of the models can pose challenges in terms of computational time and processing power. Assigning accurate material properties to the finite elements is critical for realistic simulations. Variability in bone density and composition can affect the accuracy of the FEA results, requiring careful calibration and validation. Its integration with ML promises improved predictive accuracy, and efforts are underway to apply FEA to in vivo imaging for non-invasive assessment of bone strength [190]. This approach can potentially revolutionise clinicians’ evaluation and monitoring of bone health [191]. Finite element analysis (FEA) enhances the capability of bone micro-CT analyses by providing detailed mechanical insights into bone microarchitecture. By simulating real-world loading conditions, FEA predicts bone strength and fracture risk, offering a comprehensive understanding of bone health. This combination of high-resolution imaging and advanced computational modelling positions FEA as a critical tool in research and clinical settings, driving innovations in osteoporosis management, implant design, and personalised medicine. As technology advances, integrating FEA with other diagnostic tools promises to revolutionise bone health assessments and treatment strategies further. Beyond biomechanical modelling, FEA-derived insights have direct clinical implications for surgical practice. In osteoporotic bone, simulations can inform the decision to augment implant fixation with bone cement, whereas cement-free prostheses may be preferred in patients with sufficient bone stock. Furthermore, FEA-based density maps can support optimal screw placement in regions of higher bone strength, thereby enhancing implant anchorage and stability. These applications illustrate how FEA not only advances biomechanical understanding but also contributes to improved operative outcomes [143,192,193,194].

Standard FEA workflow (from scan to quantitative metrics). (1) Image acquisition: obtain high-resolution scans (micro-CT ex vivo; HR-pQCT in vivo) with appropriate voxel size and field of view for the anatomical site [177,178,179,180,181]. (2) Preprocessing: apply noise reduction and motion/beam-hardening correction; perform isotropic resampling and (if needed) registration across time points [177]. (3) Segmentation: delineate cortical and trabecular compartments; derive density maps or binarised masks that preserve thin trabeculae [178,179,180,181]. (4) Mesh generation: construct voxel-based hexahedral meshes (direct voxel-to-element) or surface-based tetrahedral meshes for complex geometries [177,178,179,180,181]. (5) Material mapping: assign element-wise elastic (and, if relevant, plastic/ductile) properties using density–elasticity relationships or tissue-level vBMD; incorporate anisotropy when available [177,178,179]. (6) Boundary conditions and loads: specify physiologic scenarios (axial compression, bending, torsion); constrain DOFs to avoid rigid-body motion; use site-specific load cases (e.g., distal radius fall) [182,183,184,185,186]. (7) Solver and criteria: compute apparent stiffness/modulus and estimate failure load using established criteria (e.g., strain-based thresholds such as the Pistoia criterion) [182,183,184,185,186,190,191]. (8) Validation and sensitivity: compare predictions with experiments (mechanical testing, phantom studies); perform mesh-density and parameter sensitivity checks [182,183,184,185,186]. (9) Outputs and reporting: report stiffness, apparent modulus, failure load, and spatial maps of strain/stress; document meshing strategy, boundary conditions, and material laws to ensure reproducibility [190,191]. Concrete example. In HR-pQCT of the distal radius, a voxel-based FEA pipeline maps vBMD to element-wise elastic moduli, applies a uniaxial compression surrogate of a fall on the outstretched hand, and outputs apparent stiffness and failure load; these values are then integrated with clinical covariates to stratify incident fracture risk [182,183,184,185,186,190,191].

#### Beyond Strength: Transport and Poromechanics from Microstructure

Beyond estimating mechanical competence, microstructure-derived computational models are increasingly used to study transport phenomena within trabecular bone. Using μCT-based geometries, finite-element and related approaches can simulate advection–diffusion of solutes, estimate permeability from pore-space morphology, and couple fluid and solid behaviour via poroelastic or fluid–structure interaction (FSI) frameworks. Such models probe local mass transport and shear fields that influence osteocyte signalling, remodelling, and drug penetration. Recent work has, for example, leveraged FEM to simulate Brownian motion of tracer particles directly within human trabecular networks segmented from μCT, linking pore topology to transport behaviour and providing microstructurally grounded diffusivity estimates (e.g., [195]). Extending FE pipelines to include advection–diffusion–reaction terms, permeability tensors, and appropriate boundary conditions (pressure, flow, or mixed) enables quantitative hypotheses about nutrient delivery, mechanotransduction, and antibiotic access in diseased bone. Taken together, these transport-oriented simulations complement strength-oriented FEA by connecting form to function along both mechanical and biophysical axes, thereby broadening the translational utility of μCT/HR-pQCT-based models in orthopaedics and precision therapeutics. Practical note. These transport/poromechanics models can be co-registered to strength-oriented FEAs on the same μCT volume, enabling multi-physics assessment (e.g., relating regions of high strain to predicted perfusion pathways) without additional scanning [195].

### 3.2. Artificial Intelligence in HR-pQCT

Machine learning (including CNNs) enhances HR-pQCT by automating artefact correction, bone structure classification, and predictive risk modelling. These approaches improve diagnostic accuracy, reduce manual effort, and support personalised treatment [7,174,175]. Moreover, machine learning models can be integrated with HR-pQCT data to develop predictive models for bone health, aiding in personalised treatment strategies and risk assessments [176,196]. Moreover, artificial intelligence (AI) can integrate HR-pQCT data into predictive models for bone health, assisting in the development of personalised treatment strategies and risk assessments [174].

Future integration of imaging, genomics, and AI could identify novel biomarkers and optimise treatment strategies [197]. HR-pQCT offers complementary strengths over imaging modalities, such as MRI and ultrasound, in evaluating bone quality and fracture risk. While MRI provides superior soft tissue contrast and ultrasound offers radiation-free assessments, HR-pQCT excels in visualising the detailed microarchitecture of bone tissue. The high resolution of HR-pQCT is unrivalled when evaluating trabecular and cortical microstructures, which are crucial for assessing fracture risk [133]. Despite advancements, challenges remain: cost, accessibility, speed, and the need for standardised protocols [174,197]. The evolution from pQCT to HR-pQCT marks a significant leap in the ability to assess bone health, offering detailed insights into bone microarchitecture and strength. As technological advancements unfold, HR-pQCT is poised to play an increasingly crucial role in diagnosing and managing bone disorders. By addressing current limitations and integrating with other cutting-edge technologies, HR-pQCT can revolutionise bone health care and research, ultimately improving patient outcomes.

Concrete AI examples (HR-pQCT and micro-CT): (i) HR-pQCT motion-artefact correction: a convolutional neural network trained on paired low-/high-motion scans suppresses blur and ringing, improving downstream morphometrics and FEA-derived failure load estimates [175,176,196,198,199]. (ii) HR-pQCT cortical porosity mapping: U-Net–style CNNs segment the cortical shell and intra-cortical pores to compute cortical porosity and thickness; these features, combined with age/clinical variables, feed gradient-boosting or logistic models for incident-fracture prediction [200,201,202,203,204,205,206]. (iii) Micro-CT segmentation for FE meshing at scale: 3D CNNs automate trabecular/cortical segmentation on large ex vivo datasets, stabilising thickness/connectivity metrics and generating high-fidelity masks for voxel-based FE models; this reduces observer variability and accelerates end-to-end strength analysis [207,208]. Together, these examples illustrate how AI either improves input data quality (denoising, artefact reduction) or directly derives quantitative biomarkers (e.g., cortical porosity) that integrate with or augment FEA for quantitative bone analysis.

## 4. Discussion

Bone health underpins quality of life by preventing injury and maintaining mobility. Disorders such as osteoporosis reduce bone mass and increase fragility, creating urgent diagnostic and therapeutic challenges. High-resolution imaging technologies, notably micro-CT and HR-pQCT, have become indispensable for assessing bone structure. However, their roles differ: micro-CT remains a research gold standard, while HR-pQCT offers a clinically feasible approach [30,31,32]. This technology is crucial for studying bone morphology and has applications in medicine, industry, and geology. Micro-CT’s ability to visualise 3D structures facilitates comprehensive analyses of complex biological specimens, making it a standard tool in bone tissue research [11,209,210,211]. While versatile and used beyond bone (e.g., soft tissue, developmental biology), its main limitation is accessibility: high cost, specialised infrastructure, and limited standardisation across platforms. For instance, micro-CT can map neuronal architecture, analyse meat quality in food science, and investigate the effects of bone ageing [10,47,48,49,67]. Its ability to generate 3D anatomical data makes it invaluable in biomedical research, enhancing our understanding of biological structures and functions [63,173]. Integrating HR-pQCT data with finite element analysis (FEA) enables biomechanical evaluations of bone strength, thereby further enhancing our understanding of bone health [174,176]. FEA adds biomechanical context to imaging by predicting load response and fracture risk. Its strength lies in personalised modelling, but outputs depend heavily on segmentation quality and assumptions about material properties, which vary across populations and scanners. This integration is crucial for developing personalised treatment plans for at-risk populations, aiding in osteoporosis management and implant design [177,178,179,187,188,189,212,213]. AI/ML enhances HR-pQCT analysis by automating artefact correction and enabling predictive risk modelling. While promising, current models are often trained on limited datasets and lack external validation. Regulatory approval and integration into clinical workflows remain open challenges [174,176,196]. Moreover, high-resolution imaging data open perspectives for the patient-specific design of scaffolds. Additive manufacturing techniques could be used to fabricate implants with precisely tailored trabecular densities, optimised to bear local mechanical loads and potentially supporting faster osseointegration. Although bone will eventually undergo natural remodelling processes, this approach may enhance initial stability and integration [214,215]. Another aspect that has not been strongly emphasised is the detailed understanding of bone remodelling and growth. By combining longitudinal micro-CT/HR-pQCT studies with mechanistic modelling, future research could provide deeper insights into the dynamics of bone turnover and implant integration [216]. The fidelity of μCT/HR-pQCT-based models depends critically on upstream imaging and modelling choices. First, segmentation thresholds and partial-volume effects can shift BV/TV, Tb.Th, Ct.Po, and SMI, with cumulative impact on stiffness and failure surrogates; SMI, in particular, is sensitive to resolution and binarisation and warrants cautious interpretation in vivo. Second, image resolution and mesh size dictate whether thin trabeculae and intracortical pores are resolved; mesh-convergence checks are essential. Third, boundary conditions (e.g., platen, kinematic, or physiologic multi-directional constraints) substantially influence apparent moduli and local stress/strain fields. Fourth, material laws and mapping—linear elastic vs. nonlinear, isotropic vs. orthotropic, and tissue- vs. apparent-level density–modulus relationships—alter predicted responses; assumptions should be stated and, where possible, validated. Finally, load case representativeness (activities of daily living, implant-specific scenarios) and motion artefacts in in vivo HR-pQCT contribute additional uncertainty. Transparent reporting and standardised pipelines help maintain comparability and clinical relevance.

## 5. Conclusions

High-resolution imaging is transforming bone health research and clinical diagnostics. Key conclusions are as follows:

1. Micro-CT remains the research gold standard, providing unparalleled resolution for ex vivo and preclinical studies, though it is limited in direct clinical use due to radiation and cost.
2. HR-pQCT is the clinical frontier, offering in vivo assessment of trabecular and cortical bone microarchitecture, but its adoption is constrained by availability, motion artefacts, and lack of standardisation.
3. FEA and ML add biomechanical and predictive layers, enhancing risk assessment and personalisation, but depend on high-quality datasets and rigorous validation.
4. Future progress hinges on accessibility, cost reduction, and protocol harmonisation to translate these advances into everyday clinical care.

Together, these tools promise earlier diagnosis, improved treatment planning, and a shift toward precision medicine in bone health.

## 6. Future Perspectives

### 6.1. Multimodal Imaging

Future research will increasingly rely on the integration of micro-CT, HR-pQCT, MRI, PET, and optical imaging, allowing complementary insights into bone microarchitecture, metabolism, and tissue composition. A major challenge is the harmonisation of data formats, workflows, and clinical protocols across modalities.

### 6.2. Contrast Agents

Targeted contrast agents hold promise for visualising bone turnover, microdamage, and inflammation. However, their widespread adoption will depend on safety validation, regulatory approval, and cost-effectiveness.

### 6.3. Artificial Intelligence and Machine Learning

AI and ML will play growing roles in automated segmentation, artefact reduction, and predictive modelling, reducing operator dependence. Yet, clinical translation requires large-scale datasets, external validation, interpretability, and regulatory acceptance.

### 6.4. Three-Dimensional Printing and Regenerative Medicine

High-resolution imaging already supports 3D printing of patient-specific implants. Future directions include optimising scaffolds and monitoring regenerative therapies. Key barriers remain cost and workflow integration.

### 6.5. Global Health and Accessibility

Portable imaging devices, combined with AI-based cloud analysis, could expand access to osteoporosis and fracture diagnostics in low-resource settings. To succeed, solutions must be affordable, robust, and supported by training and infrastructure.

### 6.6. Standardisation and Translation

Broad clinical adoption will require standardised protocols, analysis pipelines, and reporting frameworks. International consensus initiatives are essential to ensure comparability, reproducibility, and regulatory approval.

## Figures and Tables

**Table 1:** Cross-modality comparison for bone and musculoskeletal imaging. Voxel sizes and representative use cases compiled from [6,72,78,81,82,83].

Modality	Typical Spatial Resolution	Contrast Mechanism	Ionising Radiation	Soft-Tissue Contrast	3D Bone Microarchitecture (Trabeculae/Cortex)	In Vivo Suitability	Typical Bone Uses	Key Limitations
Micro-CT (lab, ex vivo)	~1–10 µm voxels (sub-µm with nano-CT)	X-ray attenuation (calibratable to mineral density)	Yes (specimen only)	Limited without contrast agents	Excellent (quantitative trabecular/cortical metrics; FE models)	Ex vivo; small-animal in vivo variants exist	Morphometry, porosity, BMD, implants/scaffolds, digital histology, FE	Dose/scan time; FoV and specimen-size constraints; beam hardening
SR micro-CT (ex vivo)	Sub-µm	Monochromatic X-ray attenuation	Yes (beamline)	Limited without contrast	Excellent (ultra-high res)	Ex vivo	Lacuno-canalicular network, micro-damage, mineral mapping	Facility access; sample size limits
HR-pQCT (clinical in vivo)	61–82 µm	X-ray attenuation	Very low effective dose	Limited soft tissue	Good (coarser than micro-CT; clinical in vivo)	Yes (peripheral sites)	Osteoporosis assessment, longitudinal monitoring, FE strength	Peripheral only; motion; partial-volume at trabecular scale
MRI (clinical)	~0.2–1.0 mm (sequence-dependent)	Proton density and relaxation; excellent soft tissue	No	Excellent (marrow, cartilage, synovium)	Limited (UTE/ZTE capture cortex signal; not trabecular morphometry at clinical voxel sizes)	Yes	Marrow composition, oedema, cartilage, soft tissue around bone	Lower mineral sensitivity; long scans; artefacts with metal
PET-CT	PET: ~4–6 mm; CT: 0.5–1 mm	Molecular radiotracer uptake + CT anatomy	Yes (radiotracer + CT)	PET indirect; CT limited	Limited (microarchitecture below PET resolution)	Yes	Turnover, infection/inflammation, oncology; CT for localization	Radiation; cost; limited spatial detail of trabeculae
Ultrasound (MSK)	~0.1–0.3 mm (high-freq probes)	Acoustic impedance	No	Good for superficial soft tissue	No intraosseous (cortex blocks beam)	Yes	Tendons/ligaments, cortical surface, guidance	Operator-dependent; cannot image through cortex

**Table 2:** pQCT parameters with associated abbreviations, descriptions, and units—data adapted from [111,162].

Metric Measures	Abbreviation	Description	Standard Unit	Recommended Variable for
Bone volume ratio	BV/TV	Ratio of bone volume to total volume in the ROI	%	trabecular bone
Cortical bone area	Ct.Ar	Cortical bone area	mm^2^	cortical bone
Total cross-sectional area	Tt.Ar	Area inside the periosteal envelope	mm^2^	cortical bone
Cortical area fraction	Ct.Ar/Tt.Ar	Ratio of cortical bone area to total cross-sectional area	%	cortical bone
Cortical thickness	Ct.Th	Average cortical thickness	mm	cortical bone
Cortical porosity	Ct.Po	Relative voxel-based measure of the volume of the intracortical pore space normalised by the sum of the pore and cortical bone volume	%	cortical bone
Trabecular separation	Tb.Sp	Mean distance between trabeculae	mm	trabecular bone
Trabecular thickness	Tb.Th	Mean thickness of trabeculae	mm	trabecular bone
Trabecular number	Tb.N	Mean number of trabeculae per unit length	mm^−1^	trabecular bone

## Data Availability

The original contributions presented in this study are included in the article. Further inquiries can be directed to the corresponding author.
